# Supplementary material for: From sequence to enzyme mechanism using multi-label machine learning
Source: BMC Bioinformatics. 2014 May 19;15:150. doi: 10.1186/1471-2105-15-150 (PMC4229970; doi:10.1186/1471-2105-15-150)
Supplement: Additional file 2 — Java code of ml2db. Additional file ml2db_code.tar.gz contains the Java source code to run the multi-label machine learning experiments and save the results to database. The code’s Javadoc is included. [file 1471-2105-15-150-S2.zip › additional file 2/ml2db/ml2db/doc/mulan/transformations/multiclass/class-use/SelectionType.html]

Uses of Class mulan.transformations.multiclass.SelectionType


JavaScript is disabled on your browser.


- Overview
- Package
- Class
- Use
- Tree
- Deprecated
- Index
- Help

- Prev
- Next

- Frames
- No Frames

- All Classes

## Uses of Class mulan.transformations.multiclass.SelectionType

- Packages that use SelectionType

  | Package | Description |
  | --- | --- |
  |  |  |
  | --- | --- |
  | mulan.transformations.multiclass |  |
- - ### Uses of SelectionType in mulan.transformations.multiclass

    Methods in mulan.transformations.multiclass that return SelectionType

    | Modifier and Type | Method and Description |
    | --- | --- |
    |  |  |
    | --- | --- |
    | `static SelectionType` | SelectionType.`valueOf(java.lang.String name)` Returns the enum constant of this type with the specified name. |
    | `static SelectionType[]` | SelectionType.`values()` Returns an array containing the constants of this enum type, in the order they are declared. |

    Constructors in mulan.transformations.multiclass with parameters of type SelectionType

    Constructor and Description

    `SelectBasedOnFrequency(SelectionType type)`

    Initializes the transformation with a `SelectionType`

- Overview
- Package
- Class
- Use
- Tree
- Deprecated
- Index
- Help

- Prev
- Next

- Frames
- No Frames

- All Classes
